# A Feasibility Study on Noninvasive Blood Glucose Estimation Using Machine Learning Analysis of Near-Infrared Spectroscopy Data

**DOI:** 10.3390/bios15110711

**Published:** 2025-10-25

**Authors:** Tae Wuk Bae, Byoung Ik Kim, Kee Koo Kwon, Kwang Yong Kim

**Affiliations:** 1 Daegu-Gyeongbuk Research Center, Electronics and Telecommunications Research Institute, Daegu 42994, Republic of Korea; 2 Electronic Advanced Development Team, AJIN Industrial Co., Ltd., Gyeongsan 38462, Republic of Korea

**Keywords:** blood glucose, near-infrared, reflectance, regression, multilayer perceptron

## Abstract

This study explored the feasibility of noninvasive blood glucose (BG) estimation using near-infrared (NIR) spectroscopy with dog blood samples. A sensor module employing three representative wavelengths (770 nm, 850 nm, and 970 nm) was tested on an artificial blood vessel (ABV) and a thin pig skin (TPS) model. BG concentrations were adjusted through dilution and enrichment with injection-grade water and glucose solution, and reference values were obtained from three commercial invasive glucometers. Correlations between NIR spectral responses and glucose variations were quantitatively evaluated using linear, multiple, partial least squares (PLS), logistic regression, regularized linear models, and multilayer perceptron (MLP) analysis. The results revealed distinct negative correlations at 850 nm and 970 nm, identifying these wavelengths as promising candidates for noninvasive glucose sensing. Furthermore, an NIR–glucose database generated from actual dog blood was established, which may serve as a valuable resource for the development of future noninvasive glucose monitoring systems.

## 1. Introduction

Diabetes mellitus is a metabolic disorder caused by insulin deficiency and insulin resistance. In all types of diabetes, chronic hyperglycemia can lead to various health complications, including heart attack, congenital defects, neurological damage, renal failure, and blindness. Conversely, hypoglycemia, characterized by abnormally low BG levels, may cause coma, confusion, and even death. The global prevalence of diabetes was estimated at 30 million individuals in 1985 and increased to 150 million by 2000. According to the International Diabetes Federation (IDF), approximately 371 million people were living with diabetes by the end of 2012, and this number is projected to reach 552 million by 2030 [1].

The total blood volume in the human body consists of approximately 55% plasma, 45% erythrocytes, and less than 1% buffy coat (comprising leukocytes and platelets). Glucose in plasma is transported through the arteries and delivered to the capillaries via the arterioles of the circulatory system. It has been reported that glucose concentrations in arterial and capillary blood are nearly identical, whereas venous BG may be slightly lower [2]. Upon entering the capillaries, glucose diffuses into the interstitial fluid surrounding tissue cells. Compared with arterial and capillary glucose levels, interstitial glucose exhibits a physiological delay of approximately 5–15 min. Traditional finger-prick BG monitoring primarily collects capillary blood, with small contributions from arterioles and venules. In contrast, noninvasive approaches detect glucose within the interstitial fluid; however, these measurements reflect plasma glucose trends with a physiological time delay, which should be carefully considered.

Various approaches for efficient BG measurement have been extensively studied [3,4,5,6,7,8]. These methods are generally classified into invasive, minimally invasive, and noninvasive techniques [3]. Invasive methods directly measure BG by obtaining blood samples, typically from the fingertip. Minimally invasive methods employ subcutaneous sensors to assess glucose concentration in the interstitial fluid (ISF). In contrast, in vivo noninvasive methods estimate BG levels indirectly without direct blood sampling. These methods are commonly divided into transdermal and optical techniques. Transdermal approaches include impedance spectroscopy, sonophoresis, and reverse iontophoresis. Optical approaches include near-infrared (NIR), mid-infrared (MIR), and long-infrared (LIR) spectroscopy, photoacoustic spectroscopy, Raman spectroscopy, occlusion spectroscopy, and optical polarimetry.

The key optical properties that have recently attracted attention for glucose detection include the glucose absorption coefficient, specific optical rotation, and Raman shift. Sensors that detect the interaction between light and glucose rely on changes in NIR/MIR absorption, the angle of optical rotation, and the intensity of Raman signals [6]. Optical polarimetry utilizes the optical activity of glucose, specifically its stable optical rotation. However, its accuracy in glucose monitoring is limited by factors such as light scattering in the skin, linear birefringence in the dermis, corneal birefringence in the eye, and the presence of other optically active biomolecules in blood [7]. Raman spectroscopy, based on the Raman scattering effect, enables quantitative analysis of glucose concentration; nevertheless, the inherently weak Raman scattering cross-section of glucose often results in poor signal strength. Furthermore, the Raman spectrum of glucose can be easily obscured by strong background noise from surrounding tissues and fluids.

Unlike the aforementioned techniques, infrared (IR) spectroscopy has recently been extensively studied because it does not require expensive measurement devices and can be implemented in compact forms. To exploit the absorption bands of glucose in the 750–1400 nm IR region, light-emitting diodes (LEDs) with NIR wavelengths of 660 nm, 850 nm, and 940 nm are commonly employed [9]. Haxha et al. [10] monitored variations in glucose concentration using a 940 nm LED. In this region, hemoglobin is the dominant absorber; thus, fluctuations in blood oxygen saturation may distort the measurement results [11]. The use of an 850 nm LED is particularly advantageous because an isosbestic point of oxyhemoglobin and deoxyhemoglobin occurs at approximately 800 nm [12]. At the isosbestic point, hemoglobin absorption remains constant regardless of oxygen saturation. This wavelength is therefore applied to account for hemoglobin absorption changes when evaluating glucose variations at the same spectral range.

In early NIR approaches, Haxha et al. [10] did not specify the detailed wavelength ranges employed in their experiments, focusing only on the correlation between sensor output and glucose concentration, without demonstrating clinical accuracy or reproducibility. Narkhede et al. [13] presented the correlation between glucose concentration and reflected signal intensity using a single 940 nm LED-based reflection sensor; however, they did not sufficiently address limitations such as low signal-to-noise ratio and susceptibility to interference from other tissue components and external factors. Segman [14] did not provide detailed spectral data or analysis results, instead emphasizing the clinical performance evaluation of the device. Joshi et al. [15] proposed the iGLU 2.0, an IoMT-based wearable system intended for commercialization; however, the prototype was bulky and unsuitable for continuous wearable use. Although accuracy metrics such as mean absolute deviation (MAD), root mean squared error (RMSE), and Clarke Error Grid (CEG) were reported, the effects of absorption and reflectance at 940 nm and absorption at 1030 nm on glucose estimation were not analyzed. Rajeswari and Vijayakumar [16] integrated an NIR sensor system with a data analysis framework, demonstrating practical applicability across different skin types. Nevertheless, despite employing a single wavelength at 940 nm and various machine learning techniques to calculate accuracy metrics such as MAE, MSE, and CEG, they did not analyze the spectral characteristics of IR wavelengths related to glucose. A recent review [17] also highlighted that most existing studies have mainly focused on proposed devices and accuracy evaluations, with limited investigation of the underlying spectral analysis of the optical components employed.

Recent studies on noninvasive BG monitoring have increasingly utilized photoplethysmography (PPG) [18,19,20]. Yen et al. [18] applied neural networks to PPG signals and achieved high-accuracy glucose prediction, while Alghlayini and Marzuki [19] and Hossain et al. [20] effectively combined convolutional neural networks with PPG signals for glucose estimation. However, these studies primarily focused on validating the accuracy between actual and predicted glucose values, without providing sufficient quantitative evidence on the direct correlation between glucose concentration and PPG signals. Moreover, most experiments were conducted with a limited number of subjects under controlled environments, which poses challenges for practical commercialization. Thus, although the technological potential of PPG-based approaches is high, large-scale clinical data and standardized validation protocols are essential to ensure medical reliability.

In this study, we aim to provide quantitative data analysis and clear correlations between actual BG concentrations and infrared optical signals. We conduct an in-depth investigation of how variations in glucose concentration influence infrared optical responses, with the goal of enhancing the accuracy and reliability of noninvasive glucose monitoring systems. Ultimately, this work is expected to contribute to the future clinical applicability of noninvasive glucose estimation.

## 2. Materials and Methods

### 2.1. NIR Sensor Modules Used in Experiments

**Figure 1:**
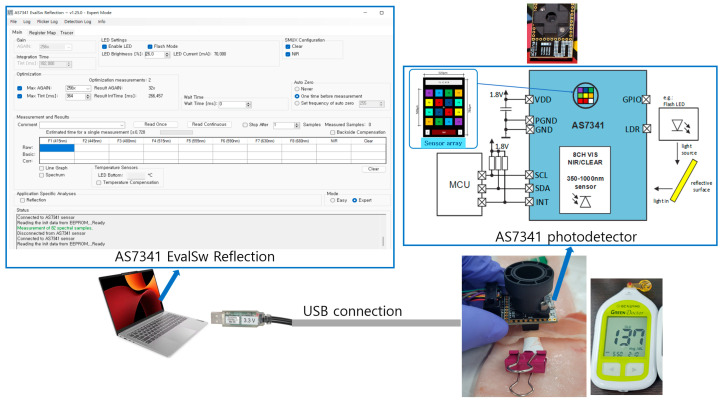
Experimental environment using AS7341 photodetector.

The photodetector employed in this study was the AS7341, an 11-channel spectrometer IC manufactured by AMS (Premstaetten, Austria) [21]. The experimental environment using the AS7341 photodetector is shown in Figure 1. In the AS7341 photodetector, light reflected or transmitted from the LED is incident on the sensor array located at the center-top of the IC. The sensor integrates eight channels for visible light, one channel for NIR, one clear channel without an optical filter, and a dedicated channel for ambient light flicker detection. Furthermore, six independent 16-bit ADC channels are embedded for parallel data processing. Device control and spectral data access are realized via a serial I^2^C interface. The AS7341 photodetector was connected to a laptop via USB, and the brightness and driving current of the LED were controlled using the evaluation software.

**Figure 2:**
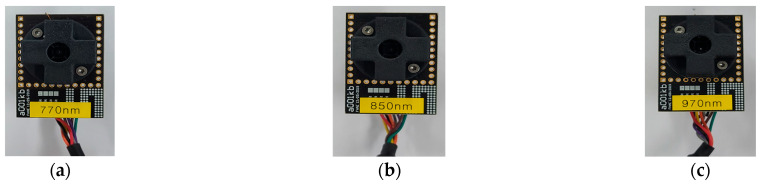
Three types of NIR sensor modules developed using the AS7341 photodetector for BG response analysis: (**a**) 770 nm, (**b**) 850 nm, and (**c**) 970 nm.

To analyze the infrared response characteristics related to BG, three types of NIR sensor modules (770 nm, 850 nm, and 970 nm) were developed by employing the AS7341 photodetector, a compact reflective multi-spectral sensor, as illustrated in Figure 2. Specifically, the infrared LEDs in the NIR sensor modules were replaced with wavelengths corresponding to the spectral regions suitable for BG measurement experiments. In the selection of near-infrared LEDs, we chose representative wavelengths within the 750–1400 nm range, where the absorption characteristics of water and glucose are prominent. The 770 nm wavelength was included as a reference, since it corresponds to a spectral region without a notable glucose absorption peak and thus serves as a baseline for comparison. Additionally, in this study, the 770 nm wavelength was used solely as a baseline reference due to its negligible glucose-specific absorption. No subtraction or division operation was applied between the 770 nm signal and other wavelengths; instead, it served only to verify that the variations observed at 850 nm and 970 nm were attributable to glucose concentration changes.

### 2.2. Experimental Setup and Procedure

**Figure 3:**
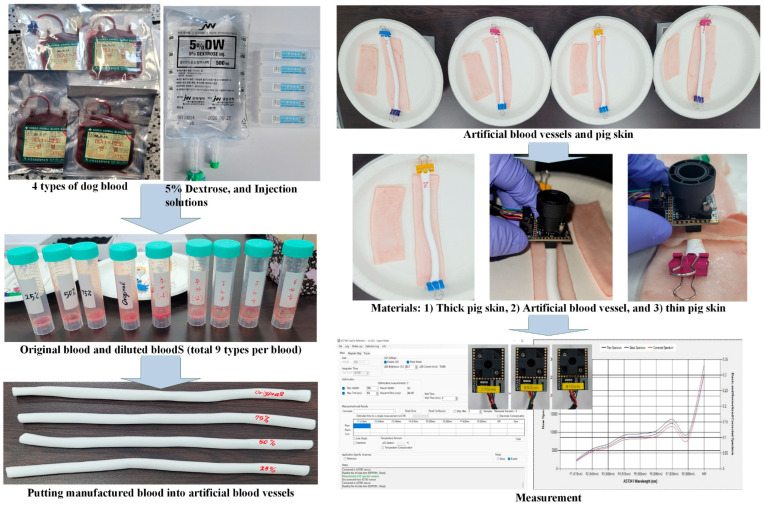
Experimental procedure.

The experimental procedure is shown in Figure 3. For the experiments, four samples of canine blood were purchased from the Korea Animal Blood Bank [22]. To increase the variety of blood concentrations through dilution, 5% dextrose injection solution (CJ CheilJedang, Seoul, Korea) and sterile water for injection (Huons Co., Seongnam, Korea) were employed. Diluted blood samples were prepared by adding sterile water to whole blood, while concentrated blood samples were produced by supplementing with 5% dextrose solution. A total of 36 blood samples were prepared to cover a glucose concentration range of approximately 0 mg/dL to 600 mg/dL, determined based on the maximum measurable range of the invasive glucose meters used in the experiment. Each sample was diluted or concentrated by adding sterile water or 5% glucose solution to whole blood using a pipette. To ensure sample quality, the prepared blood was thoroughly mixed, and glucose levels of all samples were measured using multiple commercial glucose meters to verify that the target concentrations were achieved.

The purpose of this study was to examine the relationship between optical properties and controlled glucose concentrations. It should be noted that the intrinsic physiological regulatory mechanisms of glucose—such as tissue distribution, metabolism, and excretion—were not represented in this experimental model [23]. Each sample was injected into an artificial blood vessel (ABV). The ABVs, manufactured by Wellcron Co., Daegu, Korea, had an inner diameter of 6 mm and a wall thickness of 850 µm.

Two experimental models were considered: (1) the ABV model, in which only the experimental blood was injected into the ABV, and (2) the TPS model, in which a thin layer of pig skin was placed on the ABV. In both models, porcine skin was present at the bottom of the structure. For these two cases, the three types of developed NIR sensor modules were applied to measure the infrared reflectance characteristics associated with BG levels.

In this study, we simulated the in vivo vascular and skin environment using a dual-layer model composed of ABV and TPS layer placed on top of it. This configuration was designed to reproduce the glucose measurement conditions in the human body in a simplified manner, allowing for quantitative analysis of NIR reflectance characteristics under stable conditions as the glucose concentration varied. The TPS closely resembles human skin both structurally and biochemically [24], and its near-infrared spectroscopic properties—namely absorption and scattering spectra—have been reported to be similar to those of human tissue [25]. The ABV, on the other hand, is a tubular structure made of a material similar to that of blood vessels, into which dog blood was circulated to mimic subcutaneous blood flow. Recent studies have also developed skin phantoms containing artificial vessels to validate the performance of non-invasive glucose sensors [23,26]. However, in actual in vivo conditions, factors such as vascular distribution, hemodynamics, and surrounding tissue interference are far more complex than those represented by our experimental model. The present setup reflects glucose concentration changes in capillary or arterial blood without incorporating pulsatile components, and therefore, discrepancies may exist when compared with interstitial fluid-based measurements in real human applications.

In the software configuration of the NIR sensor modules, the LEDs were operated in the default “Enable mode.” Since the on/off state of the flash did not have a significant effect on the infrared reflection response characteristics, it was set to “Off.” The illuminance conditions during measurement were maintained within the range of 1–2 lux, and the LED current values used to control LED brightness are summarized in Table 1.

**Figure 4:**
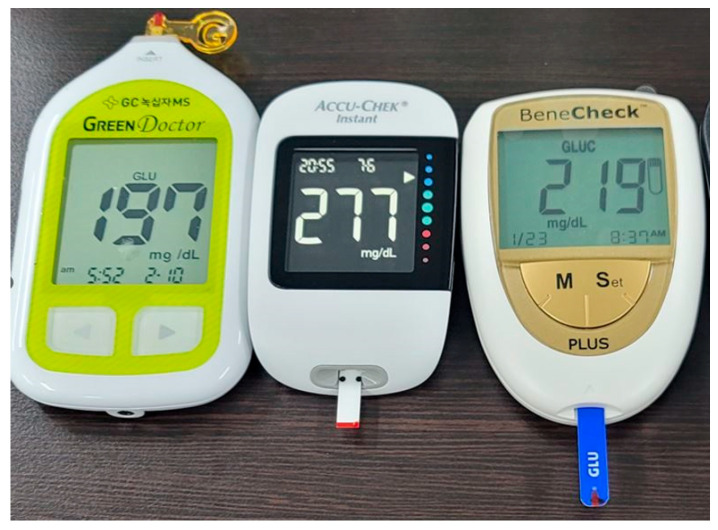
Various invasive glucometers employed in the experiments.

To measure the reference BG levels, three types of invasive glucometers were used, as shown in Figure 4: GreenDoctor (GCMS, Ansan, Korea), AccuChek (Roche Diagnostics, Seoul, Korea), and BeneCheck (CS MediTech, Seoul, Korea). Among these, GreenDoctor was the most frequently employed in this study, while AccuChek is known to provide the highest accuracy. Although there were differences in the absolute glucose values obtained from each device, the overall trends in glucose measurements were found to be consistent across the glucometers.

### 2.3. NIR–Glucose Database and Analysis Methods

Using three types of invasive glucometers, 36 experimental blood samples, and three NIR wavelengths, NIR–glucose data were collected for both the ABV model and the TPS model. In this study, various analytical techniques were applied, including linear, multiple, partial least squares (PLS), logistic regression, regularized linear models, and multilayer perceptron (MLP) neural networks. The linear-type models were employed to investigate the primary (first-order) relationship between glucose concentration and NIR reflectance, chosen for their intuitive interpretability. PLS regression, a standard method in NIR spectroscopy studies, was adopted because it effectively mitigates multicollinearity and provides stable performance even with a relatively small dataset. Additionally, regularized linear models—Lasso, Ridge, and Elastic Net—were utilized for their ability to perform variable selection and prevent overfitting. These models allow the inclusion of all three NIR reflectance features while automatically reducing the influence of less relevant variables. Meanwhile, an MLP neural network was introduced to explore potential nonlinear relationships. Considering the limited dataset size, the MLP architecture was designed as a lightweight model with a single hidden layer consisting of five nodes.

## 3. Results

### 3.1. Linear Analysis of NIR–BG Data for ABV Model

To evaluate the feasibility of noninvasive BG monitoring based on NIR spectroscopy, linear regression analyses were performed between the glucose values measured by three commercial invasive glucometers and the NIR reflectance values obtained from the ABV model. The NIR reflectance values were measured at 770 nm, 850 nm, and 970 nm, which are representative wavelengths widely used in noninvasive optical sensing due to their relevance to the absorption characteristics of water and glucose [27,28].

**Figure 5:**
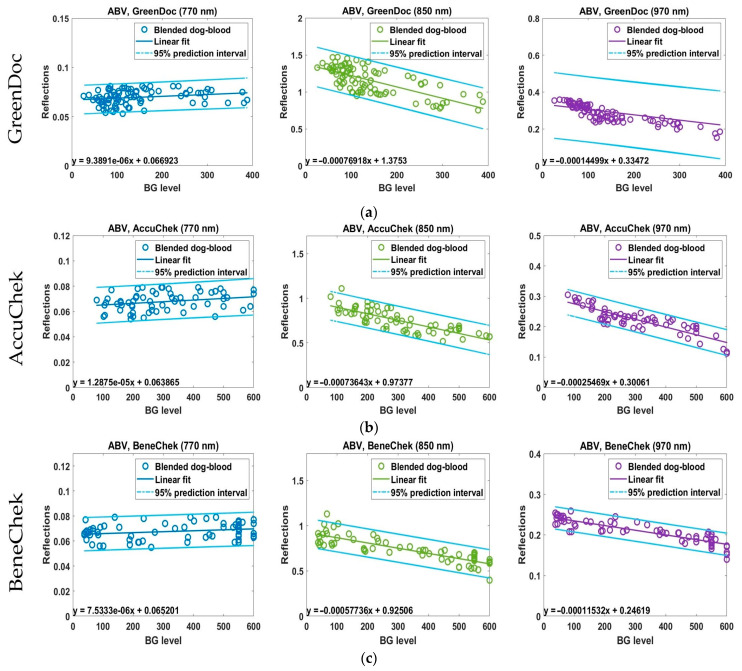
Linear regression plots of NIR–BG data for the ABV model using invasive glucometers: (**a**) GreenDoc, (**b**) AccuChek, and (**c**) BeneCheck.

Figure 5 presents the linear regression plots of the NIR–BG data for the ABV model using each invasive glucometer. In each plot, the horizontal axis represents the blood glucose (BG) levels measured by the glucometers, while the vertical axis indicates the corresponding NIR reflectance values. Each graph includes a first-order linear regression line fitted using the “polyfit” function in MATLAB 2024, along with the regression equation and the 95% prediction interval [29]. These plots are structured to provide an intuitive understanding of the glucose sensitivity of NIR reflectance according to both device type and wavelength. The visual representation of the regression equations highlights the differences in wavelength-dependent sensitivity and emphasizes the importance of wavelength selection in noninvasive glucose monitoring technology, as demonstrated in the ABV experiments.

In the ABV experiments, the infrared reflectance values exhibited a slightly proportional relationship with blood glucose at 770 nm, whereas inverse relationships were observed at 850 nm and 970 nm. According to previous reports, blood glucose and NIR reflectance are theoretically expected to show an inverse correlation [30]. GreenDoctor showed the most distinct response at 850 nm; however, at 970 nm, the regression slope was nearly zero, indicating markedly reduced sensitivity. In other words, at 850 nm, the regression line demonstrated a strong decrease in reflectance with increasing BG, while at 970 nm the decrease was relatively weak.

AccuChek displayed strong negative slopes at both 850 nm and 970 nm, with the steepest slope at 850 nm, thereby exhibiting the highest linear sensitivity in ABV-based optical glucose measurements. BeneCheck also showed negative correlations at 850 nm and 970 nm, while at 770 nm a slight positive slope was observed. The vertical spread of NIR reflectance values was approximately 0.03 at 770 nm, 0.7 at 850 nm, and 0.1 at 970 nm, confirming that the 850 nm wavelength produced the widest distribution of reflectance values for a given glucose level. Moreover, compared with 970 nm, the negative slope of the NIR–BG regression at 850 nm was more pronounced.

These findings demonstrate the potential to enhance the accuracy of noninvasive BG monitoring through optimization of wavelength–device combinations. In particular, the 850 nm band showed stable responsiveness across all devices, suggesting that it may serve as a primary target for future calibration models.

As shown in Table 2, the linear regression models presented in Figure 4 were analyzed using the “fitlm” function in MATLAB 2024 [31]. In the coefficient properties, *Estimate* represents the estimated value of the coefficient for each corresponding term in the model, while *Intercept* denotes the estimated value of the constant term. The Standard Error (SE) indicates the standard error of the coefficient estimates. The tStat refers to the *t*-statistic for each coefficient, which tests the null hypothesis that “the coefficient equals zero” against the alternative hypothesis that “the coefficient does not equal zero,” given the other predictors in the model. Notably, *tStat* is calculated as Estimate/SE.

The *tStat* indicates how far the estimated coefficient is from zero under the null hypothesis. A larger absolute value of *tStat* implies that the corresponding coefficient is more likely to be statistically significant, whereas a smaller absolute value suggests insignificance. Based on the *tStat* value and the degrees of freedom, the probability of observing such a *t* value by chance is calculated as the *p*-value. Thus, a large |*tStat*| corresponds to a small *p*-value and statistical significance, while a small |*tStat*| corresponds to a large *p*-value and statistical insignificance.

The *p*-value represents the probability value associated with the *t*-statistic for a two-sided hypothesis test, as well as the *p*-value of the *F*-test for the overall model. If the *p*-value of a specific term’s *t*-statistic exceeds 0.05, it indicates that the term is not significant at the 5% significance level when considering the other predictors in the model (whereas a value ≤ 0.05 indicates statistical significance).

The RMSE estimates the standard deviation of the error distribution, while *R*^2^ represents the coefficient of determination. The *R*^2^ value indicates the percentage of variability in the response variable (i.e., BG) that is explained by the model. In other words, *R*^2^ reflects the explanatory power of the model, with values closer to “1” indicating higher predictive accuracy. RMSE reflects the prediction error, and lower values correspond to better predictive performance.

The analysis revealed that AccuChek and BeneCheck exhibited similar wavelength-dependent characteristics. Both devices showed significant and strong negative linear correlations between BG and NIR reflectance at 850 nm and 970 nm. For both devices, the *R*^2^ values tended to increase with wavelength, reaching the highest linearity at 970 nm (*R^2^* = 0.7618 for AccuChek and *R*^2^ = 0.7633 for BeneCheck). In contrast, at 770 nm, the R^2^ values were as low as 0.0666 and 0.0552, respectively, indicating very limited linearity and low significance between reflectance and BG. In terms of sensitivity, the absolute slopes were maximal at 850 nm (AccuChek: −7.36 × 10^−4^; BeneCheck: −5.77 × 10^−4^), suggesting that the response of the glucometers to reflectance changes was greatest at this wavelength. Across all three wavelengths, AccuChek showed statistical significance with *p* < 0.05, whereas BeneCheck did not reach significance at 770 nm (*p* ≈ 0.064). These findings suggest that the ABV model effectively simulates in vivo optical conditions and supports its utility as a valid evaluation tool for the development of noninvasive glucose monitoring technologies.

In the case of GreenDoc, a distinct trend was observed compared to the other two devices. The highest coefficient of determination was obtained at 850 nm (*R*^2^ = 0.5050), whereas markedly lower values were recorded at 770 nm (*R*^2^ = 0.0486) and 970 nm (*R*^2^ = 0.0757). Notably, at 970 nm, the linearity deteriorated substantially, in contrast to the other devices. The sensitivity was also highest at 850 nm (−7.70 × 10^−4^), and statistical significance was confirmed across all three wavelengths (*p* < 0.05). Although the explanatory power at 770 nm was limited, the regression coefficient of the GreenDoc device exhibited a directional trend consistent with that of the other devices.

All three devices exhibited a negative slope at 850 nm, indicating that an increase in reflectance was associated with a decrease in BG levels. In contrast, the absolute slope values at 770 nm were very small, with low *R*^2^ values, suggesting that this wavelength provides limited information for glucose estimation. These findings highlight the importance of device-specific strategies for optimal wavelength selection. For AccuChek and BeneChek, the use of 970 nm is advantageous for ensuring higher explanatory power in single-wavelength measurements, whereas combining 850 nm (for enhanced sensitivity) and 970 nm (for improved linearity) is effective in multi-wavelength approaches. In contrast, GreenDoc showed the best performance at 850 nm, making this wavelength the most appropriate basis for measurement and calibration strategies. Furthermore, since 770 nm exhibited low explanatory power across all devices and, in some cases, did not reach statistical significance, it is advisable to exclude this wavelength from analysis or assign it a lower weighting in model development.

### 3.2. Linear Analysis of NIR–BG Data for TPS Model

**Figure 6:**
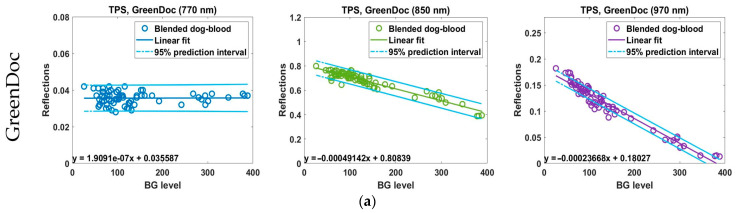

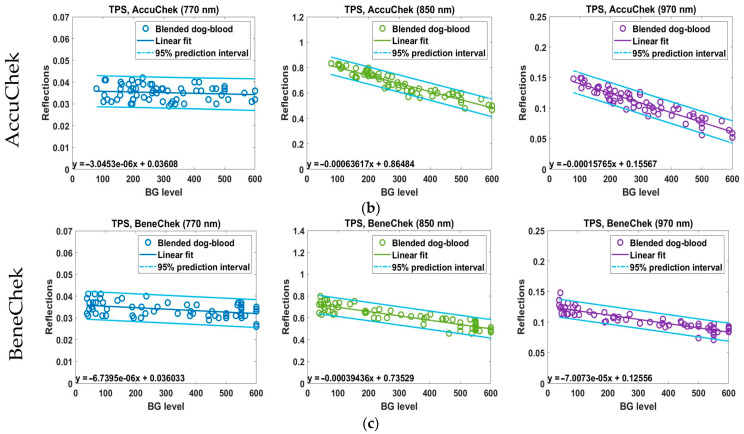
Linear regression plots of NIR-BG data obtained using TPS and the invasive glucose meters: (**a**) GreenDoc, (**b**) AccuCheck, and (**c**) BeneCheck.

Figure 6 presents the linear regression plots between the BG values measured by the invasive glucose meters and the NIR reflectance values for TPS. In the case of GreenDoc, a small positive slope was observed at 770 nm, which gradually shifted to negative slopes at 850 nm and 970 nm, indicating a decrease in NIR reflectance with increasing glucose concentration as the wavelength increased. AccuChek consistently exhibited negative slopes across all wavelengths, with the highest sensitivity and relatively good linearity at 850 nm. Similarly, BeneChek also showed negative slopes across all wavelengths, with the strongest linearity at 850 nm. Overall, the TPS-based results demonstrated greater variability in device- and wavelength-dependent linear responsiveness compared to the ABV-based results. In general, linearity was lower and noise levels were higher, a trend that was particularly pronounced for BeneChek. These findings suggest that the scattering and absorption effects in tissue-based models are more complex, highlighting the need for more sophisticated calibration models or device-specific optimization. Nevertheless, as in the ABV experiments, all invasive glucose meters exhibited strong negative slopes and high negative linear sensitivity at both 850 nm and 970 nm.

As illustrated in Figure 6 and summarized in Table 3, the linear regression analysis between NIR reflectance and invasive glucose values obtained from the TPS experiments revealed device- and wavelength-specific differences in explanatory power. GreenDoc exhibited the best linear fit at 970 nm (*R*^2^ = 0.944, slope = −2.37 × 10^−4^, *p* = 1.48 × 10^−45^), with similarly high explanatory power at 850 nm (*R*^2^ = 0.900, slope = −4.91 × 10^−4^, *p* = 1.02 × 10^−36^). In contrast, no significant correlation was observed at 770 nm (*R*^2^ = 0.00009, *p* = 0.936). AccuChek achieved its highest performance at 850 nm (*R*^2^ = 0.882, slope = −6.36 × 10^−4^, *p* = 5.77 × 10^−30^), while 970 nm also maintained strong explanatory power (*R*^2^ = 0.870, slope = −1.58 × 10^−4^, *p* = 1.15 × 10^−28^). However, no statistically significant relationship was observed at 770 nm (*R*^2^ = 0.015, *p* = 0.333). For BeneChek, the strongest explanatory power was obtained at 970 nm (*R*^2^ = 0.805, slope = −7.01 × 10^−5^, *p* = 2.56 × 10^−23^), with a comparably significant correlation also observed at 850 nm (*R*^2^ = 0.798, slope = −3.94 × 10^−4^, *p* = 6.87 × 10^−23^).

It is noteworthy that, although all devices exhibited the lowest RMSE values at 770 nm (RMSE ≈ 0.0031~0.0035), this result merely reflects slopes close to zero, indicating little to no predictive power for actual BG estimation. This finding emphasizes that RMSE alone cannot be used to assess the validity of a regression model; rather, the significance of both *R*^2^ and the regression slope must be considered together. Furthermore, the intercept values varied distinctly with wavelength, ranging from 0.73 to 0.86 at 850 nm and from 0.13 to 0.18 at 970 nm, reflecting a shift in the baseline reflectance level.

These results suggest that, under TPS conditions, an increase in BG is linearly associated with a decrease in NIR reflectance, with the optimal detection wavelength being 970 nm for GreenDoc, 850 nm for AccuChek, and 970 nm for BeneChek. This indicates that differences in sensor characteristics and optical signal processing across devices are reflected in wavelength selection and explanatory power. Therefore, the present findings support that the choice of wavelength tailored to device-specific properties is critical for effective utilization of NIR spectroscopy in non-invasive BG measurement.

### 3.3. Comparison of ABV and TPS in Linear Regression Analysis

The comparative analysis of linear regression between NIR reflectance and invasive BG values under ABV and TPS conditions revealed device-specific differences in optimal wavelength and explanatory power. In the ABV experiments, both AccuChek and BeneChek exhibited the highest explanatory power at 970 nm (*R*^2^ = 0.762 and 0.763, respectively), whereas GreenDoc achieved its maximum at 850 nm (*R*^2^ = 0.505). In contrast, under TPS conditions, AccuChek performed best at 850 nm (*R*^2^ = 0.882), while both BeneChek and GreenDoc showed stronger explanatory power at 970 nm (*R*^2^ = 0.805 and 0.944, respectively).

These results demonstrate that the optimal detection wavelength may vary depending on material properties. In the TPS experiments, 970 nm was clearly superior for GreenDoc, 850 nm for AccuChek, and 970 nm for BeneChek. In contrast, under ABV conditions, both AccuChek and BeneChek responded most strongly at 970 nm, whereas GreenDoc showed relatively better fitting at 850 nm. These findings indicate that the optical properties of tissues (i.e., vascular material versus skin thickness) influence the selection of optimal NIR wavelengths for the NIR reflectance–BG relationship, and that such effects, in combination with device-specific sensor characteristics, contribute to performance differences.

Overall, the TPS model exhibited high linearity at 850 nm and 970 nm (*R*^2^ = 0.88~0.94), whereas the ABV model showed relatively lower explanatory power with *R*^2^ values ranging from 0.07 to 0.64. In both experimental settings, consistent negative slopes were observed at 850 nm and 970 nm, indicating a decrease in reflectance with increasing BG levels, thereby confirming the spectral response associated with BG sensitivity. In contrast, at 770 nm, the *R*^2^ values remained below 0.07, and the slope directions were inconsistent, suggesting that this wavelength is unsuitable for BG estimation. Among the devices, AccuChek demonstrated stable sensitivity under both ABV and TPS conditions, while GreenDoc and BeneChek showed significantly improved performance under TPS. These findings suggest that, whereas ABV provides an optically controlled environment, TPS more closely reflects the scattering characteristics of biological tissue, thereby offering a more realistic testbed for NIR-based glucose monitoring.

### 3.4. Multiple Linear Regression Analysis of NIR–BG Data

Table 4 presents the results of multiple linear regression analyses of the NIR–BG data obtained under ABV and TPS conditions. Across all devices, 850 nm and 970 nm showed significant negative coefficients, whereas 770 nm was largely nonsignificant. In the ABV experiments, AccuChek demonstrated the strongest independent contribution at 970 nm (*β* = −2057.3, *SE* = 302.6, *t* = −6.80, *p* = 5.93 × 10^−9^, 95% CI: −2650.4 to −1464.2), while 850 nm also showed a significant negative association (*β* = −360.9, *SE* = 94.8, *t* = −3.81, *p* = 3.38 × 10^−4^, 95% CI: −546.8 to −175.1). BeneChek likewise exhibited the strongest contribution at 970 nm (*β* = −4274.3, *SE* = 601.9, *t* = −7.10, *p* = 1.83 × 10^−9^, 95% CI: −5454.1 to −3094.5), with 850 nm providing additional explanatory power (*β* = −574.2, *SE* = 118.3, *t* = −4.86, *p* = 9.21 × 10^−6^, 95% CI: −546.8 to −175.1). For GreenDoc under ABV, 850 nm emerged as the primary predictor (*β* = −618.4, *SE* = 72.2, *t* = −8.56, *p* = 3.84 × 10^−13^, 95% CI: −759.9 to −476.8), whereas 970 nm exhibited only a weak trend (*β* = −252.7, *SE* = 144.3, *t* = −1.75, *p* = 0.083).

For practical interpretation, converting the coefficients to the expected BG change per 0.01 (1%) increase in reflectance—while holding the other wavelengths constant—yields the following: under TPS, GreenDoc–970 nm predicts a −25.9 mg/dL change; AccuChek–970 nm, −26.1 mg/dL; and BeneChek–970 nm, −66.3 mg/dL. In the ABV setting, AccuChek–970 nm indicates a −20.6 mg/dL decrease. By contrast, 770 nm was nonsignificant or exhibited unstable signs in many models (e.g., positive or nonsignificant effects for ABV–AccuChek and TPS–AccuChek), indicating that it is not a reliable independent predictor.

In summary, 970 nm functioned as the most powerful independent predictor across both experimental conditions (ABV and TPS), while 850 nm consistently provided supplementary contributions. Notably, the effect of 970 nm was markedly enhanced for all devices under TPS. In contrast, 770 nm exhibited limited independent contribution in the multiple regression models, suggesting that model simplification (i.e., excluding 770 nm) or focusing on 970/850 nm may be advantageous for the optimization of non-invasive optical sensors. The intercepts represent theoretical predictions at zero reflectance and thus have limited physical interpretability; therefore, emphasis should be placed on the sign, statistical significance, and 95% confidence intervals of the coefficients.

### 3.5. Sensitivity and Specificity Analysis Based on TPS and Logistic Regression

**Figure 7:**
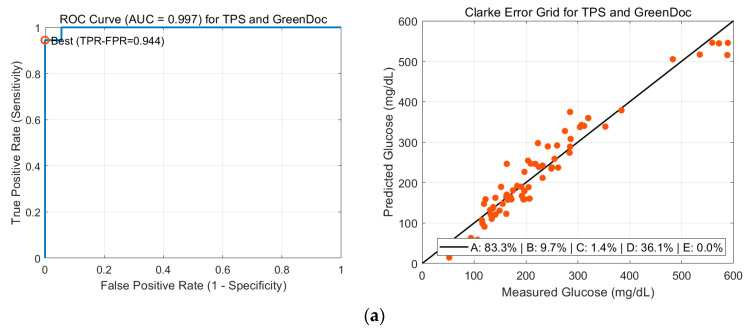

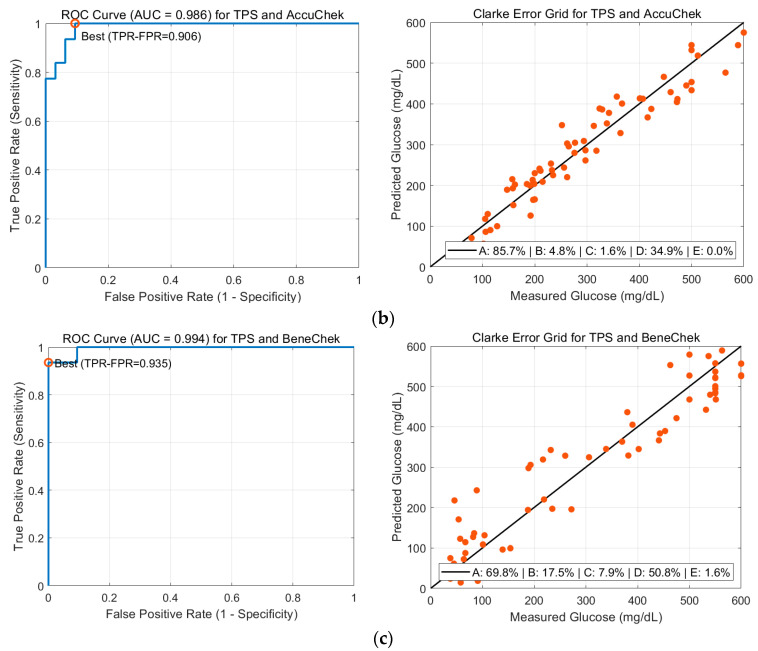
ROC and Clarke Error Grid results using logistic regression for TPS model and invasive glucose meters: (**a**) GreenDoc, (**b**) AccuCheck, and (**c**) BeneCheck.

As shown in Figure 7, the logistic regression analysis function, the “fitglm” function in MATLAB 2024, was performed to quantitatively evaluate the sensitivity and specificity of the proposed method. Using 5-fold cross-validation, we observed high classification performance for each of the three reference glucometers. For example, the logistic regression model achieved an average sensitivity of ~94.3% and specificity of ~91.4% (AUC ≈ 0.967) on the GreenDoc-referenced dataset. Similarly, for the AccuChek- and BeneChek-referenced data, the sensitivity/specificity were ~90.0%/90.0% (AUC ≈ 0.944) and ~93.3%/90.0% (AUC ≈ 0.961), respectively. These results demonstrate that the non-invasive measurement system can distinguish between different glycemic states (e.g., detecting elevated blood glucose levels) with high accuracy.

The Clarke Error Grid analysis was also conducted to assess the clinical accuracy of the predicted glucose values compared to the reference measurements. Using GreenDoc and AccuChek as references, the majority of data points fell within Zone A (approximately 83% and 85.7% of points, respectively), indicating clinically acceptable agreement, with only a small number of points in Zones B and C. However, in both cases about 35% of the points were in Zone D, representing readings with potentially significant clinical errors. When BeneChek was used as the reference, the Zone A percentage was lower (~70%), while the proportion in Zone D increased markedly to about 50.8%, indicating frequent large discrepancies between the predicted values and the BeneChek readings.

These findings suggest that the inherent error and bias of the reference invasive glucometers can significantly influence the results. Commercial glucose meters have their own accuracy limitations; for instance, the ISO 15197:2013 standard requires that 95% of readings by a home-use blood glucose system fall within ±15% of the true value (or ±15 mg/dL for low blood glucose levels). In other words, the reference devices’ readings themselves contain a certain level of error, and the apparent “accuracy” of our non-invasive predictions is ultimately bounded by the accuracy of these reference instruments. In our study, we observed that the GreenDoc meter tended to report lower glucose values overall compared to AccuChek and BeneChek, whereas BeneChek produced the highest readings. As a result, in the BeneChek-referenced data, the reference device likely overestimated glucose levels relative to the actual values, leading to larger apparent errors in the predictions. Consistent with this, as shown Table 5, the model using GreenDoc as reference achieved the best correlation (*R*^2^ ≈ 0.968, RMSE ≈ 32.16 mg/dL), whereas using BeneChek yielded a lower correlation (*R*^2^ ≈ 0.89, RMSE ≈ 70.46 mg/dL). Likewise, the Clarke error grid for the BeneChek case showed a substantially higher proportion of points in Zone D. By employing multiple glucometers and cross-validating across them, we aimed to mitigate bias from any single device, and we stress that the limitations of the reference devices must be taken into account when interpreting the results. This underscores that in evaluating a non-invasive glucose monitoring system, if an absolute ground-truth reference is absent, the measurement error of the reference device can confound the performance assessment.

Additionally, Table 4 and Table 5 employ different evaluation metrics because they correspond to different modeling approaches. Table 4 presents linear regression results (using MATLAB fitlm for continuous prediction), whereas Table 5 reports classification performance (sensitivity and specificity from a logistic regression using fitglm with 5-fold cross-validation). These analyses serve distinct purposes (continuous estimation vs. binary classification), so their results are not directly comparable.

### 3.6. Analysis Results Based on TPS Model and PLS Regression, Regularized Linear Models

**Figure 8:**
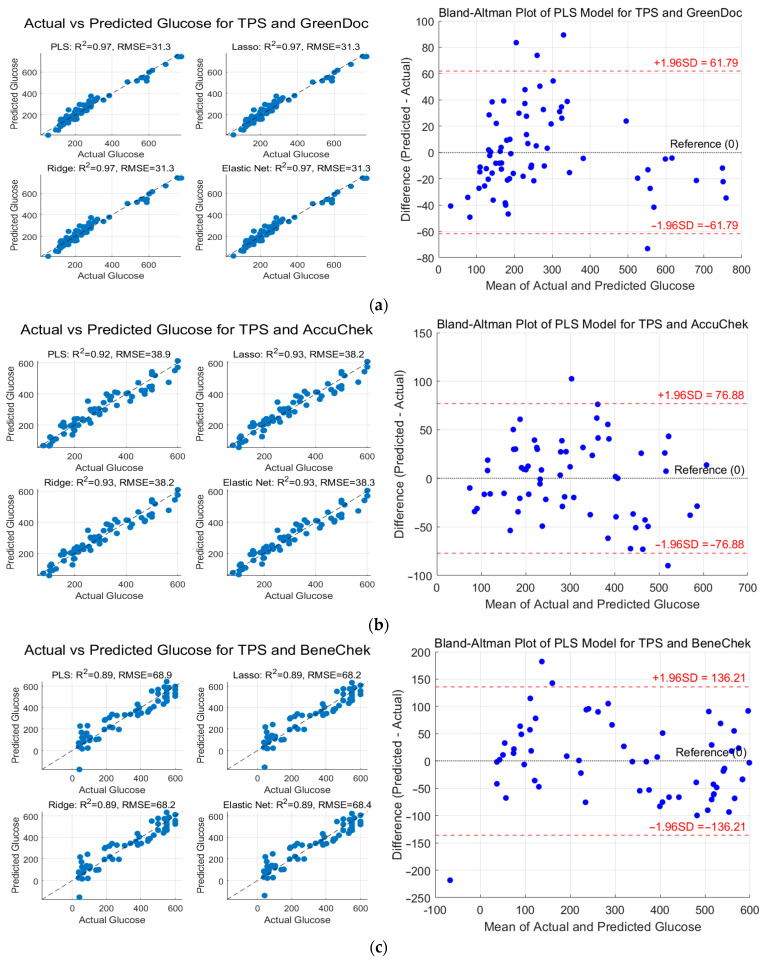
Scatter plots and Bland–Altman plots of PLS regression and regularized linear model results for the TPS model and invasive glucose meters at three wavelengths: (**a**) GreenDoc, (**b**) AccuCheck, and (**c**) BeneCheck.

Figure 8 presents the scatter plots and Bland–Altman plots of PLS regression and regularized linear model results for the TPS model and invasive glucose meters. For the GreenDoc glucose meter, all models—PLS, Lasso, Ridge, and Elastic Net—showed nearly identical performance, with *R*^2^ ≈ 0.97 and RMSE ≈ 31 mg/dL. The data points were densely distributed along the ideal 1:1 diagonal line, indicating a strong agreement between predicted and actual glucose values and minimal differences in predictive performance among the models. The Lasso and Ridge regression models achieved slightly lower RMSE values compared to PLS (a reduction of approximately 0.05 mg/dL), although the improvement was negligible. Overall, the predictions based on the GreenDoc glucose meter exhibited the highest accuracy among the three tested devices.

For the AccuChek glucose meter, the Lasso and Ridge models showed slightly improved performance compared to PLS, although the overall accuracy remained similar, with *R*^2^ ≈ 0.93 and RMSE ≈ 38 mg/dL. Most data points were clustered near the ideal 1:1 diagonal line, indicating relatively accurate predictions; however, a slightly larger dispersion was observed in the scatter plot compared to the GreenDoc results. This suggests that predictions based on AccuChek data exhibited somewhat higher variability and error. Nevertheless, the performance differences among all models were minimal, with Lasso and Ridge achieving marginally higher *R*^2^ values and lower RMSEs than PLS, indicating modest improvements in predictive accuracy.

For the BeneChek glucose meter, the predictive accuracy was lower than that of the other devices, with *R*^2^ ≈ 0.89 and RMSE ≈ 68 mg/dL, and the data showed a wider dispersion. The predicted values were more broadly scattered around the ideal 1:1 diagonal line, particularly exhibiting larger prediction errors in the high-glucose range. This suggests that when using the BeneChek device, the measurement data may have had higher variability or lower accuracy, leading to increased model prediction errors. In other words, even with the same TPS data, the predictive performance varied depending on the reference glucose meter, with BeneChek yielding the largest overall error.

The figure also includes Bland–Altman plots obtained from the PLS model for each invasive glucose meter using the TPS model data. For all three glucose meters, no significant bias was observed in the PLS model predictions, as the mean differences between the predicted and actual glucose values were close to zero. However, the 95% limits of agreement (LoA) differed depending on the device: approximately ±61.8 mg/dL for GreenDoc, ±76.9 mg/dL for AccuChek, and ±136.2 mg/dL for BeneChek, indicating the widest error range for BeneChek. These results are consistent with the previous findings, suggesting that predictions based on GreenDoc data were the most stable, whereas those based on BeneChek exhibited the largest variability and errors. In summary, glucose prediction using near-infrared (NIR) reflectance data at three wavelengths showed high correlation for both the PLS and regularized linear models, with comparable performance across model types. Nevertheless, the predictive accuracy varied depending on the reference glucose meter, implying that the accuracy and consistency of the device itself significantly affected the magnitude of the prediction errors.

### 3.7. MLP Analysis for TPS-Based NIR–BG Data

**Figure 9:**
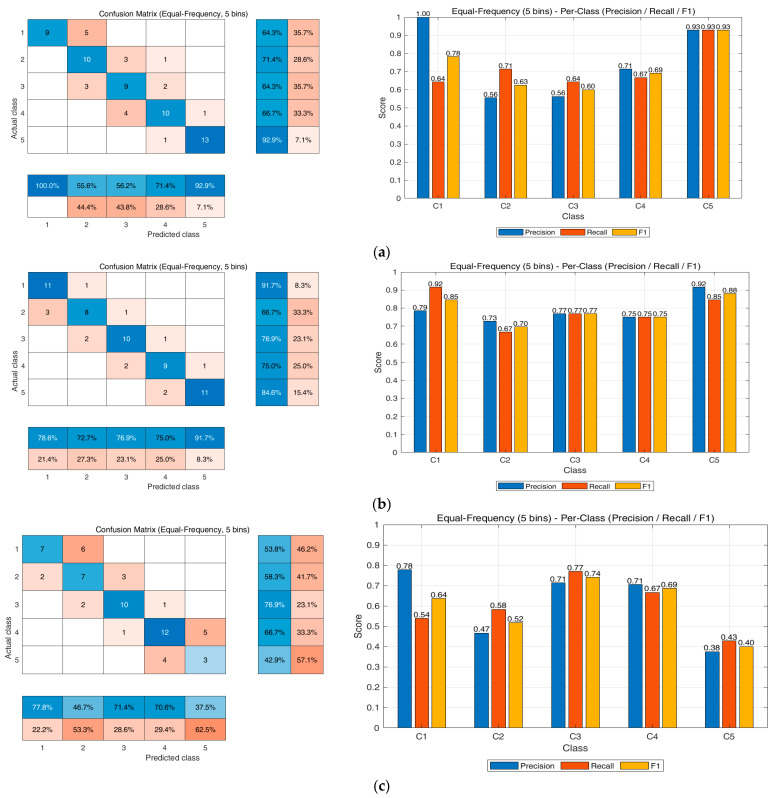
MLP training results with TPS and invasive glucose meters: (**a**) GreenDoc, (**b**) AccuChek, and (**c**) BeneChek.

Figure 9 presents the results of MLP training using NIR-BG data, with three invasive glucometers serving as reference values. An MLP was implemented in MATLAB using the “fitnet” function to predict blood glucose concentrations from three near-infrared input signals. The neural network consisted of a single hidden layer with five neurons and was trained using the Levenberg–Marquardt algorithm. The dataset was randomly divided into training (70%), validation (15%), and testing (15%) subsets, and predictions were generated from the trained network. For performance evaluation, continuous glucose values were discretized into five categories through equal-frequency binning, and classification metrics were subsequently derived based on the confusion matrix.

The GreenDoc-based model achieved an overall accuracy of 71.8%, indicating a moderate level of performance. The sensitivity values for Classes 1–5 are 0.64, 0.71, 0.64, 0.67, and 0.93, with particularly high detection capability in the hyperglycemic range (Class 5). The specificity remained above 0.86 across all classes (up to 1.00), demonstrating strong robustness against false positives. Moreover, the Precision/Recall/F1-score analysis confirmed excellent performance in Class 5 (all = 0.93), whereas reduced precision (~0.56) in Classes 2–3 highlighted class-dependent imbalance.

The AccuChek-based model achieved the highest performance among the three devices, with an overall accuracy of 76.6%. The sensitivity values for Classes 1–5 are 0.92, 0.67, 0.77, 0.75, and 0.73, respectively, with particularly strong detection capability for hypoglycemia (Class 1, sensitivity = 0.92), indicating high clinical relevance. Specificity was also stable across all classes, ranging from 0.90 to 0.98. Furthermore, Precision/Recall/F1-score analysis showed balanced outcomes across all classes, with F1-scores ranging from 0.70 to 0.88. These results suggest that the AccuChek-based model represents the most reliable reference device for training non-invasive glucose monitoring systems.

The BeneChek-based model exhibited the lowest performance among the three devices, with an overall accuracy of 54.5%. The sensitivity values across Classes 1–5 are 0.54, 0.42, 0.67, 0.62, and 0.46, with notably poor detection in Classes 2 and 5. In contrast, specificity remained relatively high, ranging from 0.76 to 0.98, indicating robustness in suppressing false positives. Precision/Recall/F1-score analysis also revealed very poor performance in Class 5 (Precision = 0.38, Recall = 0.43, F1 = 0.40), while more favorable results were observed in Classes 3 and 4, with F1-scores of 0.74 and 0.69, respectively. These results suggest that BeneChek-based measurements are subject to greater variability and underscore the need for calibration procedures to improve reliability.

Overall, the AccuChek-based model demonstrated the highest accuracy and the most balanced sensitivity and specificity, suggesting the greatest potential for clinical application. The GreenDoc-based model achieved excellent performance in a specific range (Class 5), but exhibited imbalances in the intermediate classes. The BeneChek-based model showed lower overall accuracy; however, it demonstrated limited applicability in certain ranges (Classes 3–4). These findings indicate that device-dependent variability has a significant impact on the performance of non-invasive glucose estimation models and underscore the necessity of cross-device calibration and standardization procedures to ensure reliable outcomes.

## 4. Discussion

### 4.1. Limitations of Experimental Model

In our measurement setup, pulsatile blood flow was not implemented in the experimental model; consequently, the optical signals obtained in this study represent static characteristics of arterial or capillary blood. Because the measurements were conducted under non-pulsatile, steady-state conditions, the NIR signals primarily reflect the optical properties of intravascular blood without accounting for ISF delay. Therefore, the NIR responses observed in this study differ from those typically utilized in non-invasive glucose monitoring, which are based on interstitial fluid signals (as in continuous glucose monitoring systems) or on pulsatile components such as photoplethysmographic signals. Conventional invasive glucose monitoring measures capillary BG, whereas non-invasive methods generally assess glucose levels in interstitial fluid, where a time delay of approximately 5–15 min relative to blood glucose occurs. Accordingly, the results of this study should be interpreted as optical measurements of blood signals without considering the ISF delay.

In addition, the in vitro concentration control method employed in this study represents a simplified model of the complex glucose variation that occurs in vivo. In the human body, glucose dynamically transfers from blood vessels to interstitial fluid and is consumed through insulin-mediated metabolic processes, resulting in temporal delays and spatial concentration gradients. In contrast, the present experiment intentionally excluded these physiological interactions and altered only the glucose concentration within the same blood sample. Therefore, this limitation should be taken into account when generalizing the results of this study. Future large-scale in vivo investigations are planned to further validate the findings obtained from this simplified model.

### 4.2. Lack of Sufficiently Quantified Database

A key limitation of this study lies in the lack of a large-scale, standardized database. Although a novel dataset was constructed using animal blood samples, the experimental environment and artificially controlled glucose levels restrict generalizability. In real-world clinical conditions, numerous factors such as diet, physical activity, and hormonal fluctuations influence glucose concentration and optical responses. Therefore, the establishment of large-scale, diversified, and standardized datasets is essential for the development of reliable and clinically applicable models. Such a database would form the cornerstone for training AI algorithms and ensuring their generalizability across populations.

### 4.3. Differences in Measurement Distributions of Invasive Glucometers

**Figure 10:**
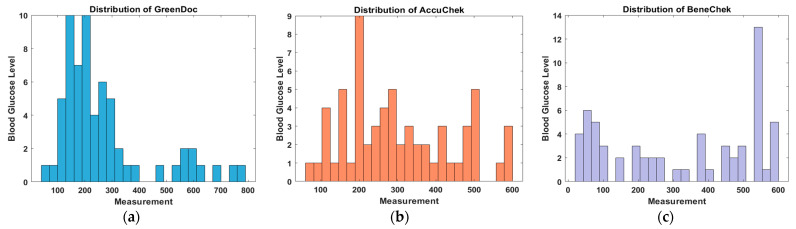
Distribution of BG measurements obtained from invasive glucometers: (**a**) GreenDoc, (**b**) AccuChek, and (**c**) BeneChek.

This study employed three invasive glucometers (GreenDoc, AccuChek, and BeneChek) as reference devices. However, notable discrepancies were observed in their measurement distributions as shown Figure 10. GreenDoc tended to underestimate glucose values, while AccuChek and BeneChek consistently produced higher readings. Such deviations may arise from differences in calibration methods, enzymatic reactions, or electrochemical sensing mechanisms [32]. Therefore, the choice of reference devices is critical in non-invasive glucose research, and cross-validation using multiple glucometers is recommended to mitigate bias and improve reliability.

### 4.4. Challenges in Developing Generalized AI Model

The predictive performance metrics reported in this study were all obtained through cross-validation on a limited dataset. Therefore, when the same models are applied to independent new samples, the actual accuracy may be lower than the results presented here. This indicates that the findings of this study should be interpreted as part of an initial exploratory stage. Future work will involve re-evaluating the model performance using a sufficiently large external validation dataset.

The MLP analysis in this study was conducted as an exploratory step for preliminary performance evaluation, given the limited number of samples. The reported accuracy metrics were obtained through cross-validation and may decrease when tested on an independent validation dataset. In future work, we plan to substantially expand the dataset and, if necessary, employ more complex models—such as those with additional hidden layers—to further improve prediction performance. And developing a universal AI model for glucose estimation remains a significant challenge. The accuracy of machine learning models is affected by inter-device variability, biological noise, and external factors such as skin pigmentation, vascular dynamics, and tissue thickness. Moreover, limited datasets often lead to overfitting and hinder generalization. Future research should therefore focus on designing adaptive AI models capable of dynamically adjusting to environmental and physiological variability, as well as leveraging transfer learning techniques to enhance model robustness across different datasets and populations.

### 4.5. Future Work

Future work will focus on expanding data collection and developing real-time monitoring systems. First, the glucose response characteristics at 770 nm, 850 nm, and 970 nm will be systematically analyzed using a wider range of animal blood samples to enhance the database. Second, advanced modeling approaches, including deep learning, will be employed to improve prediction accuracy. Third, the scope will be extended beyond glucose to include other blood biomarkers such as cholesterol and hemoglobin. Finally, efforts will be directed toward miniaturizing NIR reflective sensors for integration into wearable devices such as smart bands, thereby enabling real-time, non-invasive glucose monitoring in practical applications.

## 5. Conclusions

This study verified the feasibility of noninvasive blood glucose estimation based on near-infrared (NIR) reflectance characteristics. Across both the artificial blood vessel (ABV) and tissue-simulating pig skin (TPS) models, the 850 nm and 970 nm wavelengths showed strong negative correlations with blood glucose concentration, indicating high optical sensitivity to glucose variations. In contrast, the 770 nm wavelength exhibited negligible correlation, suggesting limited usefulness for glucose estimation. These findings highlight that wavelength selection is a key factor in determining the accuracy of optical glucose sensing and identify 850 nm and 970 nm as promising candidates for future noninvasive monitoring systems.

Various machine learning models—including linear regression, partial least squares (PLS), logistic regression, regularized linear models (Lasso, Ridge, Elastic Net), and a multilayer perceptron (MLP)—were applied for comparative analysis. All models achieved comparable predictive accuracy, confirming the robustness and consistency of the NIR–glucose relationship. Linear regression provided interpretability, PLS and regularized models ensured stable prediction with limited data, and the MLP captured nonlinear characteristics, offering potential for further accuracy improvement. This multi-model approach enhances result generalization and reliability, providing a solid foundation for developing dependable noninvasive glucose monitoring technologies.

Based on the results from the three commercial glucose meters, practical recommendations can be made according to use and environment. For clinical applications requiring high precision, AccuChek is recommended due to its superior accuracy. For home-based or preventive monitoring, GreenDoc offers a cost-effective and user-friendly option, though periodic cross-checks are advised as it tends to slightly underestimate glucose levels. BeneCheck, which showed greater variability, should be used with caution and is less suitable for critical decision-making. In summary, selecting a glucose meter should balance accuracy, usability, cost, and intended purpose, as each device has distinct advantages and limitations.

## Figures and Tables

**Table 1:** LED current values for LED brightness.

IR Wavelength	LED Brightness (%)	LED Current (mAh)
770 nm	3	12
850 nm	26	70
970 nm	3	12

**Table 2:** Linear analysis results of BG values measured by invasive glucometers and NIR reflectance in the ABV model.

Device	IR	Terms	Estimate	SE	tStat	*p*-Value	RMSE	*R* ^2^	MSE
Green- Doc	770 nm	(Intercept)	0.066923	0.0014673	45.608	5.0926 × 10^−63^			
		×1	9.3891 × 10^−6^	4.4266 × 10^−6^	2.1211	0.036728	0.00718	0.04864	0.00005
	850 nm	(Intercept)	1.3753	0.02691	51.108	3.2554 × 10^−67^			
		×1	−0.00076918	8.1181 × 10^−5^	−9.4749	4.3179 × 10^−15^	0.13175	0.50499	0.01736
	970 nm	(Intercept)	0.33472	0.0179	18.7	2.0983 × 10^−32^			
		×1	−0.00014499	5.3998 × 10^−5^	−2.685	0.0086667	0.08763	0.07572	0.00768
Accu- chek	770 nm	(Intercept)	0.063865	0.0020598	31.006	4.7866 × 10^−39^			
		×1	1.2875 × 10^−5^	6.1704 × 10^−6^	2.0865	0.041117	0.00690	0.06662	0.00005
	850 nm	(Intercept)	0.97377	0.023462	41.505	2.0067 × 10^−46^			
		×1	−0.00073643	7.0283 × 10^−5^	−10.478	2.8988 × 10^−15^	0.07857	0.64283	0.00617
	970 nm	(Intercept)	0.30061	0.0060873	49.382	6.7623 × 10^−51^			
		×1	−0.00025469	1.8236 × 10^−5^	−13.967	1.154 × 10^−20^	0.02038	0.76178	0.00042
Bene- chek	770 nm	(Intercept)	0.065201	0.0015354	42.466	5.1955 × 10^−47^			
		×1	7.5333 × 10^−6^	3.9913 × 10-06	1.8875	0.063859	0.00651	0.05518	0.00004
	850 nm	(Intercept)	0.92506	0.018096	51.119	8.6216 × 10^−52^			
		×1	−0.00057736	4.7042 × 10^−5^	−12.273	3.9874 × 10^−18^	0.07676	0.71177	0.00589
	970 nm	(Intercept)	0.24619	0.0031628	77.84	9.2512 × 10^−63^			
		×1	−0.00011532	8.222 × 10^−6^	−14.026	9.4646 × 10^−21^	0.01342	0.76332	0.00018

**Table 3:** Linear analysis results of BG values measured by invasive glucometers and NIR reflectance in the TPS model.

Device	IR	Terms	Estimate	SE	tStat	*p*-Value	RMSE	*R* ^2^	MSE
Green- Doc	770 nm	(Intercept)	0.035587	0.00076454	46.547	2.1253 × 10−54			
		×1	1.9091 × 10^−7^	2.3638 × 10^−6^	0.080764	0.93586	0.00351	0.00009	0.00001
	850 nm	(Intercept)	0.80839	0.0063348	127.61	1.2019 × 10^−84^			
		×1	−0.00049142	1.9586 × 10^−5^	−25.091	1.0235 × 10^−36^	0.02908	0.89993	0.00085
	970 nm	(Intercept)	0.18027	0.002228	80.908	6.8791 × 10^−71^			
		×1	−0.00023668	6.8886 × 10^−6^	−34.358	1.482 × 10^−45^	0.01023	0.94402	0.00010
Accu- chek	770 nm	(Intercept)	0.03608	0.0010421	34.624	8.1483 × 10^−42^			
		×1	−3.0453 × 10^−6^	3.1217 × 10^−6^	−0.97554	0.33315	0.00349	0.01536	0.00001
	850 nm	(Intercept)	0.86484	0.0099602	86.829	1.2486 × 10^−65^			
		×1	−0.00063617	2.9838 × 10^−5^	−21.321	5.7698 × 10^−30^	0.03335	0.88169	0.00111
	970 nm	(Intercept)	0.15567	0.0026103	59.636	8.5562 × 10^−56^			
		×1	−0.00015765	7.8195 × 10^−6^	−20.161	1.1524 × 10^−28^	0.00874	0.86951	0.00008
Bene- chek	770 nm	(Intercept)	0.036033	0.00073906	48.754	1.4487 × 10^−50^			
		×1	−6.7395 × 10^−6^	1.9212 × 10^−6^	−3.5079	0.0008544	0.00313	0.16786	0.00001
	850 nm	(Intercept)	0.73529	0.0097581	75.351	6.5791 × 10^−62^			
		×1	−0.00039436	2.5367 × 10^−5^	−15.546	6.8662 × 10^−23^	0.04139	0.79847	0.00171
	970 nm	(Intercept)	0.12556	0.0016994	73.885	2.1536 × 10^−61^			
		×1	−7.0073 × 10^−5^	4.4177 × 10^−6^	−15.862	2.5616 × 10^−23^	0.00721	0.80486	0.00005

**Table 4:** Multiple linear regression analysis results for NIR–BG Data.

Types of Experiments	Device	IR	Terms	Estimate	SE	tStat	*p*-Value
ABV	Green- Doc		(Intercept)	980.16	166.98	5.8698	7.975 × 10^−8^
		770 nm	×1	1341.4	1797	0.74644	0.45744
		850 nm	×2	−618.35	72.223	−8.5618	3.8375 × 10^−13^
		970 nm	×3	−252.69	144.27	−1.7515	0.083417
	Accu- chek		(Intercept)	906.12	98.589	9.1908	5.5009 × 10^−13^
		770 nm	×1	1880.5	1151.5	1.6331	0.10778
		850 nm	×2	−360.95	94.837	−3.8061	0.00033808
		970 nm	×3	−2057.3	302.62	−6.7983	5.932 × 10^−9^
	Bene- chek		(Intercept)	1445	142.96	10.108	1.719 × 10^−14^
		770 nm	×1	2890.1	1638.2	1.7642	0.082876
		850 nm	×2	−574.17	118.26	−4.855	9.2105 × 10^−6^
		970 nm	×3	−4274.3	601.94	−7.1009	1.8258 × 10^−9^
TPS	Green- Doc		(Intercept)	1040.3	59.94	17.356	1.1073 × 10^−26^
		770 nm	×1	566.17	1095.8	0.5167	0.60705
		850 nm	×2	−724.52	101.17	−7.1617	7.2484 × 10^−10^
		970 nm	×3	−2585.6	215.12	−12.019	1.7488 × 10^−18^
	Accu- chek		(Intercept)	1046	58.114	17.998	1.2232 × 10^−25^
		770 nm	×1	2589.2	1490.1	1.7376	0.087494
		850 nm	×2	−822.17	121.85	−6.7472	7.236 × 10^−9^
		970 nm	×3	−2608.5	480.69	−5.4266	1.13 × 10^−6^
	Bene- chek		(Intercept)	1743.5	95.334	18.288	5.4916 × 10^−26^
		770 nm	×1	−3495.1	2975.7	−1.1746	0.24488
		850 nm	×2	−1019.7	179.8	−5.6709	4.515 × 10^−7^
		970 nm	×3	−6626.3	945.99	−7.0045	2.6579 × 10^−9^

**Table 5:** Sensitivity and specificity results using logistic regression for TPS model and invasive glucose meters.

Types of Experiments	Logistic Regression (Threshold = Median)		5-Fold CV			Regression for CEG	
	Sensitivity (TPR)	Specificity (TNR)	Sensitivity	Specificity	AUC	*R* ^2^	RMSE
GreenDoc, TPS	0.944	0.944	0.943 ± 0.128	0.914 ± 0.078	0.967 ± 0.062	0.968	32.160 mg/dL
AccuChek, TPS	0.968	0.906	0.900 ± 0.149	0.900 ± 0.091	0.944 ± 0.109	0.926	39.487 mg/dL
BeneChek, TPS	0.935	0.969	0.933 ± 0.091	0.900 ± 0.149	0.961 ± 0.054	0.89	70.457 mg/dL

## Data Availability

The raw data supporting the conclusions of this article will be made available by the authors on request.
